# Quantitative correlation between carotid or lower limb atherosclerosis and coronary heart disease: a retrospective observational study

**DOI:** 10.3389/fendo.2025.1570942

**Published:** 2025-03-24

**Authors:** Zeyu Jiang, Shimiao Ruan, Kun Zhao, Shuhan Pan, Wenzhong Zhang

**Affiliations:** ^1^ Department of Cardiology, The Affiliated Hospital of Qingdao University, Qingdao, China; ^2^ Department of Emergency Medicine, The Affiliated Hospital of Qingdao University, Qingdao, China; ^3^ Department of Cardiology Medicine, Qingdao Central Hospital, Qingdao, China

**Keywords:** coronary heart disease, carotid atherosclerosis, lower limb atherosclerosis, ultrasound, coronary revascularization, coronary angiography

## Abstract

**Background:**

Early diagnosis and intervention are key for the treatment of coronary heart disease (CHD). Ultrasound is used to assess risk stratification in patients with coronary artery disease. However, few studies quantify the relationship between carotid or lower limb atherosclerosis and coronary revascularization. The purpose of this study is to demonstrate that the semi-quantitative degree of atherosclerosis in the neck or lower extremity vessels can predict the need for coronary revascularization, thereby establishing a predictive model for coronary revascularization based on peripheral vascular disease.

**Methods:**

Patients who underwent coronary angiography and peripheral vascular ultrasound were randomly selected for semi-quantitative analysis of the degree of coronary artery and peripheral vascular stenosis. Data from 306 patients were collected.

**Results:**

The semiquantitative score, grade score and lower limb score from vascular ultrasound were positively correlated with the Gensini score of coronary artery lesions. The semi-quantitative score (score = 2) predicted the sensitivity and specificity for coronary revascularization at 83.74% and 61.72%, respectively. The graded score (score = 2) predicted the sensitivity and specificity for coronary revascularization at 77.24% and 72.13%, respectively. The lower extremity score (score = 3) predicted the sensitivity and specificity for coronary revascularization at 90.24% and 54.55%, respectively.

**Conclusions:**

Carotid semiquantitative scores, grade scores, and lower limb scores are predictive factors for the need for coronary revascularization and can serve as auxiliary examinations for the early diagnosis of coronary artery disease.

## Introduction

As the global population ages, the mortality rate from coronary heart disease (CHD) has risen significantly, with approximately 17.9 million people dying from CHD each year, accounting for 32% of all global deaths (1). In the Asia-Pacific region, CHD remains a leading cause of morbidity and mortality, due to increased rates of smoking and drinking, poor diet, physical inactivity (2). In recent years, the early diagnosis and intervention for CHD have been the focus and priority of cardiovascular research (3). Noninvasive tests, such as coronary CT angiography (CTA), electrocardiograms and cardiac ultrasound, have been used to assess risk stratification in patients with suspected low-risk stable angina (4). As a gold standard for non-invasive examination, coronary CTA is increasingly used to rule out coronary atherosclerosis or perform risk stratification in patients with stable chest pain or acute chest pain with suspected CHD, due to its high sensitivity and specificity in detecting coronary artery disease. In the Scottish Computed Tomography of the Heart (SCOT-HEART) trial, patients who had been referred to a cardiology clinic with stable chest pain, CTA clarified the diagnosis and altered subsequent investigations and treatments (5). With the substantial advancements in coronary CTA analysis techniques, more precise quantification and characterization of atherosclerotic plaques have become possible, allowing for accurate CHD risk stratification based on imaging results (6). However, due to the high cost and the need for intravenous contrast agent injection, it is not used for population-based CHD screening. Ultrasound (US) is a safe, accurate, and low-cost noninvasive diagnostic technique that helps clinicians to quickly obtain diagnostic information (7). Echocardiography has seen significant advancements in diagnosing cardiovascular diseases. Traditional 2D echocardiography is now integrated with 3D and 4D imaging, providing more detailed views of cardiac structures and improving the assessment of ventricular function, valvular abnormalities, and myocardial perfusion (8). Doppler ultrasound allows for accurate measurement of blood flow and pressure gradients across heart chambers, which is essential for diagnosing heart failure and valvular disease. These advancements make ultrasound an increasingly important tool for both early detection and management of CHD.

Atherosclerosis is the underlying pathology for both CHD and peripheral artery disease (PAD). Previous studies have found many similarities between carotid and lower limb atherosclerosis, and coronary atherosclerosis including risk factors, clinical manifestations, pathophysiological mechanisms and treatment (9). The CAFES-CAVE study showed that the presence of arterial plaques is a strong predicter of future cardiovascular events (10). However, few studies have provided a quantitative evaluation of the relationship between carotid or lower limb atherosclerosis and coronary revascularization.

According to the guidelines of the American College of Cardiology (ACC) and the European Society of Cardiology (ESC), the assessment of patients with suspected coronary artery disease should combine clinical evaluation with noninvasive tests, incorporating advanced imaging techniques when appropriate, and conducting invasive coronary angiography for high-risk patients (11, 12). This study aligns with these recommendations by exploring the use of ultrasound to assess peripheral vascular atherosclerosis in establishing a predictive model for coronary artery reconstruction, thereby contributing to a broader framework for coronary artery disease management. This model will provide a simple, rapid, and cost-effective screening tool to determine which patients will require coronary revascularization.

## Methods

### Study population

**Figure 1:**
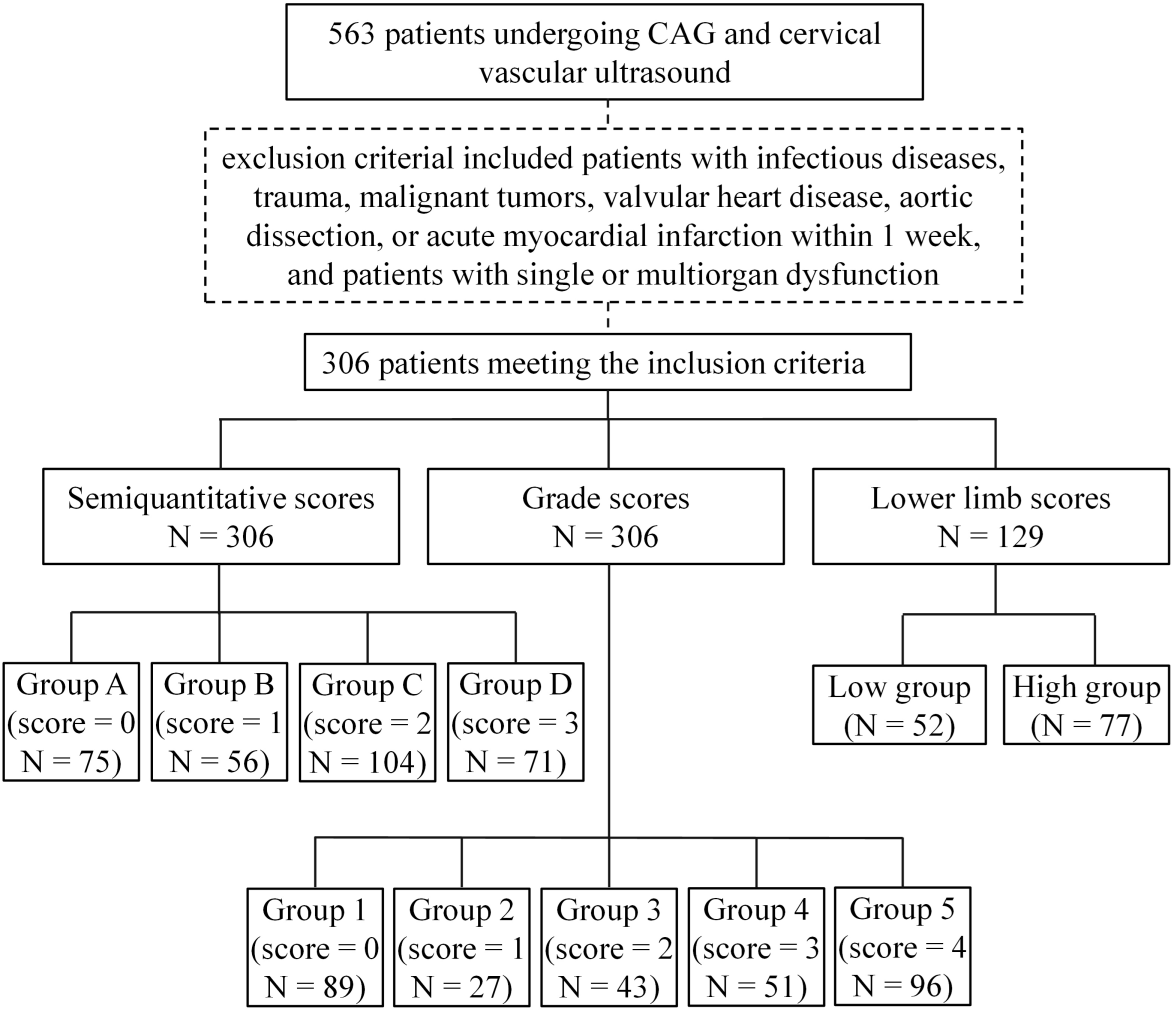
Flow diagram of patients enrollment and grouping.

We randomly selected 306 patients between 40 and 75 years who underwent coronary angiography and carotid artery ultrasound in the Department of Cardiology, Affiliated Hospital of Qingdao University from September 2020 to April 2023. Among the 306 patients, 129 also underwent lower limb vascular ultrasound. The research protocol was approved by the Ethics Committee of the Affiliated Hospital of Qingdao University (approval number: QYFY WZLL 28928, Qingdao, China), and all of the participants provided signed informed consent. The exclusion criterial included patients with infectious diseases, trauma, malignant tumors, valvular heart disease, aortic dissection, or acute myocardial infarction within 1 week, and patients with single or multiorgan dysfunction. Clinical data included sex, age, and body mass index; a history of smoking and drinking; and a diagnosis of diabetes or hypertension. Hypertension was defined as systolic blood pressure (SBP) at or above 130 mm Hg and/or diastolic blood pressure (DBP) at or above 80 mm Hg. Diabetes was defined as fasting plasma glucose (FPG): ≥126 mg/dL (7.0 mmol/L) or random plasma glucose: ≥200 mg/dL (11.1 mmol/L). Levels of total cholesterol, triglycerides, low-density lipoprotein and high-density lipoprotein were also collected. Patients were grouped according to different scoring criteria (**Figure 1**).

### Ultrasound examination

Ultrasound examinations were conducted with a DC-25 color Doppler ultrasound diagnostic system (Mindray, Shenzhen, Chian), using a 7.5 MHz vascular probe 7L4A. During the case collection process, diagnostic reports from the same experienced ultrasound physician were selected to minimize bias.

### Grading criteria for carotid or lower extremity arteries

Both the carotid and lower extremity arteries were scanned to assess the presence of plaques and the intima-media thickness (IMT). The rate of stenosis was calculated as follows: stenosis rate = (inner diameter of vessel - inner diameter of narrowest place)/inner diameter of vessel. Plaques were defined as a focal wall thickening ≥ 1.2 mm that protrude into the arterial lumen (13). The number, shape and size of the observed plaques were recorded. Semiquantitative scores, grade scores and lower limb scores were described based on plaques, IMT and stenosis rate (**Supplementary Table S1**) (14–16).

### Coronary angiography

All procedures were performed by experienced physicians via puncture of the right corpuscular artery or femoral artery in accordance with the American College of Cardiology and American Heart Association guidelines for angiography. All patients underwent a coronary angiography by an experienced physician using a Medical Angiography X-ray System (Allura-Xper-FD20, Philips Medical Systems Nederland B.V). The angiography results were evaluated using the international common diameter method. The severity of CHD was assessed using the Gensini score (17).

### Statistical analysis

The continuous variables (i.e., age, BMI (body mass index), TC (total cholesterol), TG (triglyceride), HDL (high-density lipoprotein), LDL (low-density lipoprotein) and Gensini scores) were tested for normal distribution (QQ plot, **Supplementary Figure S1**). Normally distributed variables (age, BMI, TC, LDL) are presented as means ± standard deviations, and group comparisons were performed using independent sample t-tests or analysis of variance (ANOVA). *Post-hoc* comparisons were conducted using Tukey’s test to control for multiple comparisons. For non-normally distributed variables (HDL and TG), the data was reported as median ± interquartile range, with group comparisons conducted using the Kruskal-Wallis test. Categorical variables (gender, diabetes, hypertension, smoking, drinking) were presented as a count and percentage and compared by the χ2 test. Univariate logistic regression analysis was performed to assess the associations of CHD with gender, diabetes, hypertension, smoking, drinking, age, BMI, TC, TG, HDL, LDL, Semiquantitative scores and Grade scores. Those factors with a *p*<0.05 were selected for the multivariate regression model. The receiver operating characteristic (ROC) curve, area under the curve (AUC), accuracy, and F1 score were used to evaluate the model’s predictive performance. *p* values were two-sided and indicated significance when less than 0.05. Statistical analyses were carried out with SPSS 22.0 (SPSS Inc., Chicago, IL, USA) and Python3.9.13 (Python Software Foundation, Wilmington, DE, USA).

## Results

### Correlation between carotid artery atherosclerosis and CHD

**Figure 2:**
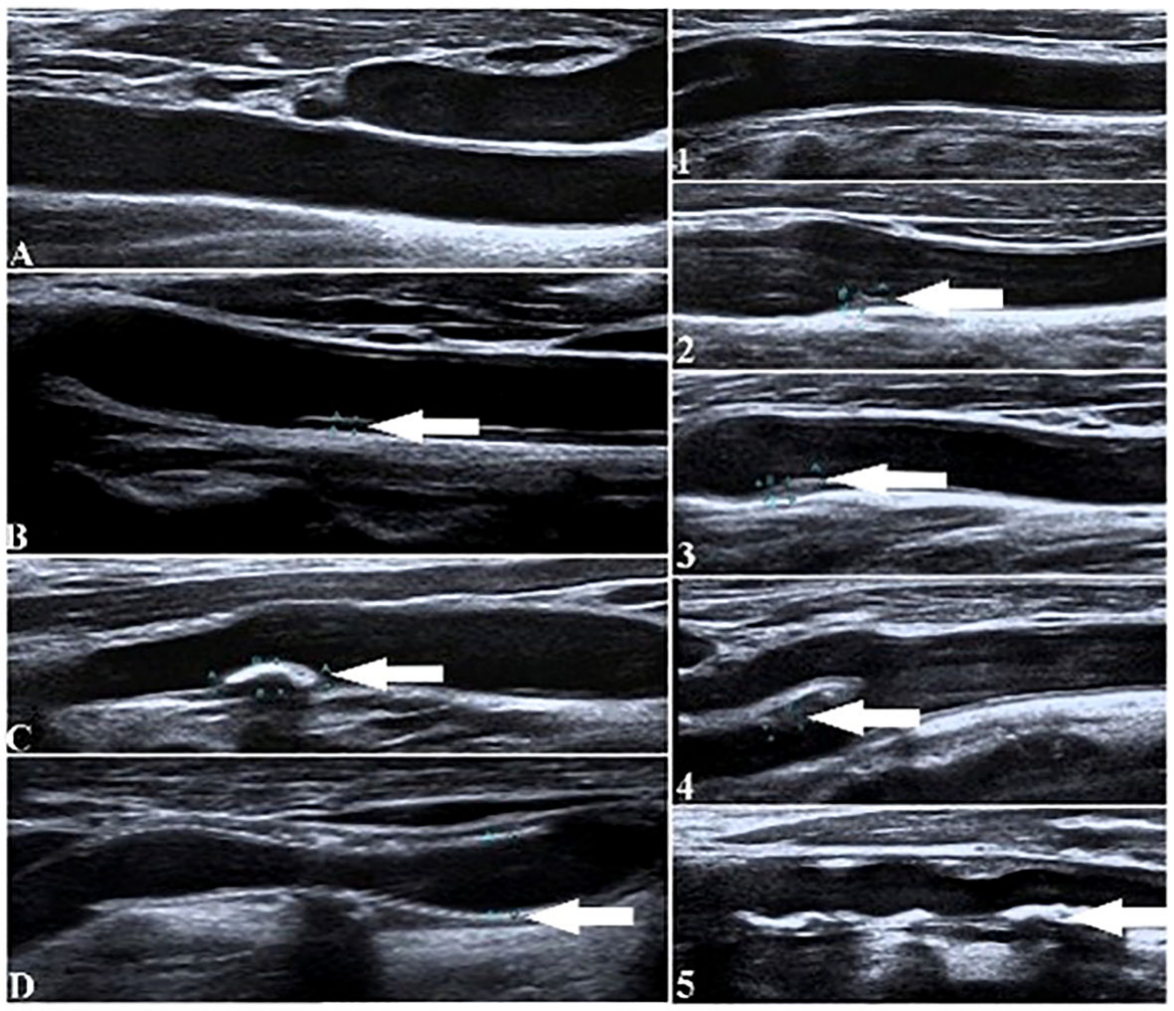
Ultrasound diagrams of neck blood vessels to show the morphology of carotid plaques under different integrations. (Left diagrams show four groups of subjects from **(A–D)** based on the semiquantitative scores. Right diagrams show the five groups of subjects based on grade scores. White arrows indicate the locations of the carotid plaques).

**Figure 3:**
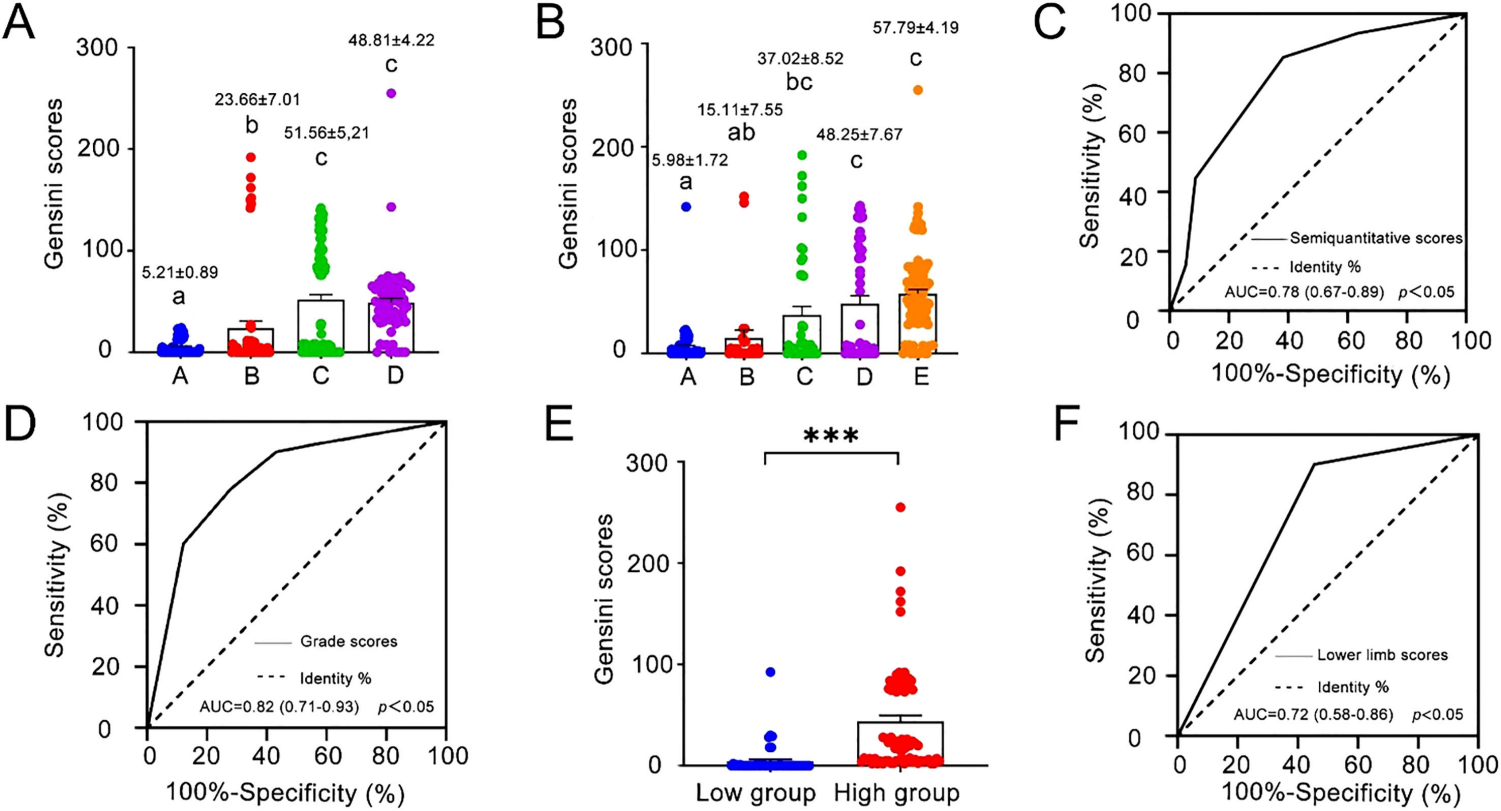
**(A)** Gensini scores for the four groups of subjects **(A-D)** based on semiquantitative scores. (Mean ± SE is presented on each column. The numbers of subjects in each group were 75 for Group A, 56 for Group B, 104 for Group C, and 71 for Group **(D)** Different lowercase letters indicate significant differences at *p* < 0.05) **(B)** Gensini scores for the five groups of subjects based on grade scores. (Mean ± SE is presented on each column. The number of subjects in each group was 89 for Group 1, 27 for Group 2, 43 for Group 3, 51 for Group 4, and 96 for Group 5. Different lowercase letters indicate significant differences at *p* < 0.05) **(C, D)** ROC curves of the semiquantitative score and the grade score for predicting the necessity of coronary revascularization. (AUC, area under the ROC curve. The sensitivity and specificity for the requirement of coronary revascularization were 83.74% and 61.72%, respectively, when the semiquantitative score was 2, and 77.24% and 72.13% when the grade score was 2) **(E)** Gensini scores for the two groups of subjects based on lower limb scores. (Mean ± SE is presented on each column. The number of subjects was 52 in the Low Group and 77 in the High Group. ***, *p* < 0.05) **(F)** ROC curve of the lower limb score for predicting the necessity of coronary revascularization. (AUC, area under the ROC curve. The sensitivity and specificity for the requirement of coronary revascularization were 90.24% and 54.55%, respectively, when the pooled scores of intimal thicknesses, plaque occurrence and stenosis ratio were higher than 3).

According to the semiquantitative scores obtained from the carotid artery ultrasound, 306 subjects were assigned to four groups, i.e., Groups A (score 0 = normal, IMT < 1.0 mm), B (score 1 = intimal thickening locally, IMT < 1.2 mm), C (score 2 = arteriosclerotic plaque formation, but no significant stenosis), and D (score 3 = stenosis rate above 25%) (**Figure 2**). There was a significant difference in average age among the groups (*F*_(3, 302)_ = 5.50, *p* = 0.001). Tukey’s test showed that the average age in Group D was significantly higher than that in Group A (*p* = 0.007). Group B had a higher percentage of females. The ratios of diabetes, hypertension and drinking in Groups B to D were larger than those of Group A (**Table 1**). The Gensini scores obtained from coronary angiography were compared among the four groups. Groups B to D had significantly higher average Gensini scores than Group A (**Figure 3A**, *F*_(3, 301)_ = 21.77, *p* < 0.0001). The average Gensini score of Group B was 4.5 times that of Group A. The average Gensini scores of Groups C and D were over 9 times that of Group A and approximately twice that of Group B.

**Table 1:** Baseline characteristics of patients in the four groups with different semiquantitative scores.

Characteristics	Group A (N=75)	Group B (N=56)	Group C (N=104)	Group D (N=71)	*p*
Female, N. (%)	20 (26.67)	27 (48.21)	32 (30.77)	21 (29.58)	0.171
Diabetes, N. (%)	14 (18.67)	19 (33.93)	42 (40.38)	18 (25.35)	0.084
Hypertension, N. (%)	32 (42.67)	32 (57.14)	56 (53.85)	38 (53.52)	0.393
Smoking, N. (%)	26 (34.67)	18 (32.14)	39 (37.50)	28 (39.44)	0.832
Drinking, N. (%)	34 (45.33)	30 (53.57)	57 (54.81)	42 (59.15)	0.135
Age, years	63.36 ± 11.22	64.48 ± 8.66	62.86 ± 9.38	68.52 ± 8.80	0.001
BMI, kg/m^2^	24.6 ± 3.22	24.42 ± 2.74	24.55 ± 2.48	25.51 ± 3.62	0.126
TC, mmol/L	4.36 ± 0.97	3.88 ± 0.97	4.15 ± 1.05	4.28 ± 1.07	0.051
TG, mmol/L	1.55 ± 0.60	1.75± 0.54	1.65± 0.73	1.81± 0.42	0.043
HDL, mmol/L	1.26± 0.48	1.19± 0.35	1.20± 0.33	1.20± 0.30	0.133
LDL, mmol/L	2.62 ± 0.74	2.32 ± 0.67	2.54 ± 0.71	2.52 ± 0.79	0.139

BMI, body mass index; TC, total cholesterol; TG, triglyceride; HDL, high-density lipoprotein; LDL, low-density lipoprotein, mmol/L.

The grade scores from the carotid artery ultrasound were used to classify the 306 subjects into Group 1 (score 0 = normal, no plaque), Group 2 (score 1 = 1 plaque with thickness ≤ 2.0 mm), Group 3 (score 2 = 2 plaques with thickness < 2.0 mm or 1 plaque with thickness > 2.0 mm), Group 4 (score 3 = 2 plaques with thickness > 2.0 mm) and Group 5 (score 4 = more than 2 plaques with thickness > 2.0 mm). Compared to Group 1, the ratios of diabetes were higher in the other four groups. Group 4 had higher ratios of hypertension and drinking, and Group 2 had higher ratios of females and smoking (**Table 2**). A significant difference in average Gensini scores existed among the five groups (**Figure 3B**, *F*_(4, 301)_ = 21.58, *p* < 0.0001). The average Gensini scores of Groups 3 to 5 were approximately 6- to 10-fold higher than that of Group 1. Although the average Gensini score of Group 2 was 2.5-fold that of Group 1, this difference was not significant in multiple comparisons using one-way ANOVA followed by Tukey’s test (*p* > 0.05).

**Table 2:** Baseline characteristics of patients in the five groups with different grade scores.

Characteristics	Group 1 (N=89)	Group 2 (N=27)	Group 3 (N=43)	Group 4 (N=51)	Group 5 (N=96)	*p*
Female, N. (%)	28 (31.46)	14 (51.85)	14 (32.56)	17 (33.33)	28 (29.17)	0.631
Diabetes, N. (%)	17 (19.10)	11 (40.74)	16 (37.20)	22 (43.14)	27 (28.13)	0.542
Hypertension, N. (%)	45 (50.56)	8 (29.63)	20 (46.51)	36 (70.59)	54 (56.25)	0.541
Smoking, N. (%)	28 (31.46)	12 (44.44)	13 (30.23)	21 (41.18)	37 (38.54)	0.529
Drinking, N. (%)	25 (28.90)	7 (25.93)	14 (32.56)	21 (41.18)	32 (33.33)	0.512
Age, years	64.0 ± 10.31	63.37 ± 9.59	61.95 ± 9.47	63.7 ± 10.15	67.10 ± 9.04	0.067
BMI, kg/m^2^	24.55 ± 3.06	24.48 ± 3.24	24.18 ± 2.59	24.71 ± 2.78	25.31 ± 3.19	0.246
TC, mmol/L	4.32 ± 0.95	4.17 ± 0.83	3.74 ± 0.91	4.40 ± 0.99	4.15 ± 1.16	0.017
TG, mmol/L	1.81 ± 0.53	1.56 ± 1.07	1.81 ± 0.71	1.78 ± 0.62	1.55± 0.52	0.513
HDL, mmol/L	1.20 ± 0.30	1.22 ± 0.39	1.17 ± 0.56	1.20 ± 0.24	1.23 ± 0.46	0.330
LDL, mmol/L	2.61 ± 0.74	2.49 ± 0.63	2.33 ± 0.61	2.60 ± 0.73	2.47 ± 0.79	0.229

BMI, body mass index; TC, total cholesterol; TG, triglyceride; HDL, high-density lipoprotein; LDL, low-density lipoprotein, mmol/L.

### Prediction of coronary revascularization by cervical vascular ultrasound

Of the 306 subjects, 123 underwent coronary revascularization surgery (surgery group) and 183 were treated with drugs (drug group), according to their coronary angiography results and the 2018 coronary revascularization guidelines (18). The ratios of females, diabetes, smoking, and drinking in the surgery group were slightly lower than those in the drug group, but the former had a larger body mass index than the latter (*p* = 0.017, df = 304) (**Table 3**). The ROC curves of the semiquantitative score and grade score were made to predict the need for coronary revascularization. The area under the ROC curve (AUC) for the correlation between semiquantitative score and coronary revascularization and between the grade score and coronary revascularization was 0.78 (95%CI: 0.74-0.84) and 0.82 (95%CI: 0.77-0.86), respectively (**Figures 3C, D**). When the semiquantitative score was 2 (Group C), i.e., the presence of carotid plaque but no significant stenosis, the sensitivity for the necessity of coronary revascularization was 83.74% (95% CI from 76.01% to 89.78%), and the specificity was 61.72% (95% CI from 54.29% to 68.82%). In contrast, when the grade score was 2 (Group 3), i.e., the presence of 2 plaques with thickness < 2.0 mm or 1 plaque with thickness > 2.0 mm, the sensitivity for the necessity of coronary revascularization was 77.24% (95% CI from 68.81% to 84.31%), and the specificity was 72.13% (95% CI from 65.04% to 78.49%).

**Table 3:** Baseline characteristics of the drug group and the surgery group in the prediction of coronary revascularization by carotid artery ultrasound.

Characteristics	Drug group (N=183)	Surgery group (N=123)	*p*
Female, N. (%)	61 (33.33)	39 (26.01)	0.667
Diabetes. N. (%)	57 (31.14)	36 (29.27)	0.727
Hypertension, N. (%)	97 (53.0)	66 (53.66)	0.911
Smoking, N. (%)	67 (31.14)	44 (24.04)	0.881
Drinking, N. (%)	62 (33.87)	37 (31.71)	0.488
Age, years	64.28 ± 10.14	65.04 ± 9.34	0.505
BMI, kg/m^2^	24.42 ± 3.03	25.26 ± 2.96	0.017
TC, mmol/L	4.20 ± 0.93	4.17 ± 1.15	0.590
TG, mmol/L	1.64 ± 0.59	1.81± 0.64	0.118
HDL, mmol/L	1.20 ± 0.41	1.21± 0.32	0.960
LDL, mmol/L	2.56 ± 0.67	2.45 ± 0.81	0.223

BMI, body mass index; TC, total cholesterol; TG, triglyceride; HDL, high-density lipoprotein; LDL, low-density lipoprotein, mmol/L.

### Correlation between lower limb vascular disease and CHD

Lower limb vascular ultrasound was performed in 129 subjects. Based on their lower limb scores, which reflected the severity of lower limb vascular lesions, they were classified into high and low groups. The high group had pooled intimal thickness, plaque occurrence and stenosis ratio scores higher than 3, and the low group had pooled scores below 3. Analysis of the basic characteristics of the two groups showed that the ratios of diabetes, smoking, and drinking in the high group were higher than those in the low group (**Table 4**). The body mass index in the high group was significantly higher than that in the low group (*p* < 0.001, df = 127), while the high-density lipoprotein level of the high group was significantly smaller than that of the low group (*p* = 0.016, df = 91.11) (**Table 4**). The average Gensini score of the high group based on coronary angiography was 10-fold that of the low group (*p* < 0.001, df = 93.27) (**Figure 3E**).

**Table 4:** Baseline characteristics of patients in the two groups with different lower limb scores.

Characteristics	Low group (N=52)	High group (N=77)	*p*
Female, N. (%)	27 (51.92)	40 (51.95)	0.407
Diabetes. N. (%)	10 (19.23)	19 (24.68)	0.471
Hypertension, N. (%)	28 (53.84)	41 (53.25)	0.947
Smoking, N. (%)	16 (30.77)	35 (45.45)	0.096
Drinking, N. (%)	14 (26.92)	27 (35.06)	0.338
Age, years	62.31 ± 10.98	63.4 ± 10.27	0.560
BMI, kg/m^2^	23.07 ± 2.56	24.98 ± 2.48	<0.001
TC, mmol/L	4.11 ± 0.56	4.21 ± 1.10	0.520
TG, mmol/L	1.72 ± 0.48	1.78 ± 0.62	0.979
HDL, mmol/L	1.20 ± 0.26	1.44 ± 1.07	<0.001
LDL, mmol/L	2.54 ± 0.43	2.53 ± 0.80	0.962

BMI, body mass index; TC, total cholesterol; TG, triglyceride; HDL, high-density lipoprotein; LDL, low-density lipoprotein, mmol/L.

### Prediction of coronary revascularization by lower limb vascular ultrasound

Of the 129 subjects undergoing lower limb vascular ultrasound, 41 underwent coronary revascularization surgery (surgery group), and 88 were treated with drugs (drug group) according to the results of the coronary angiography and the 2018 coronary revascularization guidelines. The ratios of diabetes and smoking in the surgery group were higher than those in the drug group (**Table 5**). The AUC from the ROC curve for the correlation between the lower limb score and coronary revascularization was 0.72 (95%CI: 0.64-0.79) (**Figure 3F**). When the pooled scores of intimal thicknesses, plaque occurrence and stenosis ratio were higher than 3 (the high group), the sensitivity for the necessity of coronary revascularization was 90.24% (95% CI from 77.45% to 96.14%) and the specificity was 54.55% (95% CI from 44.17% to 64.54%).

**Table 5:** Baseline characteristics of the drug group and surgery group in the prediction of coronary revascularization by lower limb vascular ultrasound.

Characteristics	Drug group (N=88)	Surgery group (N=41)	*p*
Diabetes. N. (%)	12 (13.64)	17 (41.46)	0.065
Hypertension, N. (%)	48 (54.55)	21 (51.22)	0.727
Smoking, N. (%)	32 (36.36)	19 (46.34)	0.284
Drinking, N. (%)	29 (32.95)	12 (29.27)	0.678
Age, years	62.65 ± 1.14	63.63 ± 1.61	0.622
BMI, kg/m^2^	23.95 ± 0.31	24.79 ± 0.33	0.067
TC, mmol/L	4.21 ± 0.09	4.10 ± 0.16	0.544
TG, mmol/L	1.66 ± 0.50	1.81 ± 0.71	0.821
HDL, mmol/L	1.28 ± 0.69	1.20 ± 0.21	0.091
LDL, mmol/L	2.62 ± 0.07	2.37 ± 0.11	0.057

BMI, body mass index; TC, total cholesterol; TG, triglyceride; HDL, high-density lipoprotein; LDL, low-density lipoprotein, mmol/L.

### Univariate and multivariable logistic regression analysis

**Figure 4:**
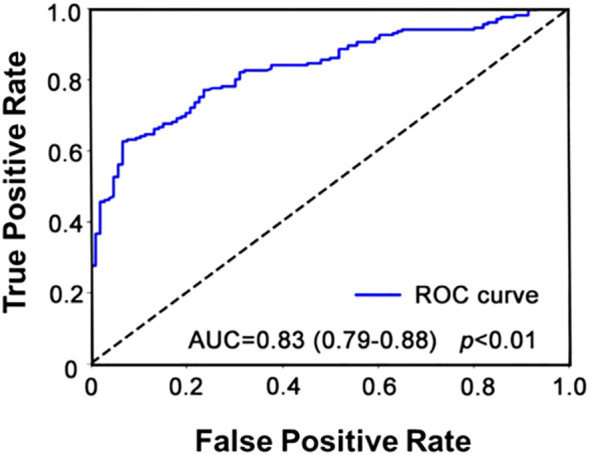
ROC curve for multivariate logistic regression analysis. The AUC was 0.83, the 95% CI was 0.79-0.88, the accuracy was 0.74, and F1 score was 0.81.

In order to control potential confounding factors, we first performed a univariate logistic regression analysis (**Supplementary Table S2**). Variables with *p*<0.05 in the univariate analysis were included in the multivariate logistic regression model (**Table 6**), and an ROC curve was generated (**Figure 4**). No multicollinearity was detected among the variables (**Supplementary Figure S2**). The Hosmer-Lemeshow test (*p*=0.801) and calibration plots indicated a good model fit (**Supplementary Figure S3**). The results indicate that HDL (OR=0.31, 95%CI: 0.16-0.59, *p*<0.001) is a protective factor for prediction of coronary revascularization, while semiquantitative scores (OR=1.98, 95%CI: 1.65-2.47, *p*<0.001) and grade scores (OR=1.95, 95%CI: 1.39-2.73, *p*<0.001) are risk factors for prediction of coronary revascularization. An increase of 1mmol/L in HDL was associated with a 69% reduction in the odds of requiring coronary revascularization, whereas a one-unit increase in the semiquantitative score and grade score was associated with a 98% and 95% increase in the odds, respectively. The area under the ROC curve of the predicted model was 0.83 (0.79-0.88), the accuracy was 0.74, and F1 score was 0.81. The results show that the model could accurately predict the need for coronary revascularization using the above scores. In the study, 129 patients underwent lower extremity vascular ultrasound. Using ANOVA and univariate logistic regression analysis, we found lower limb score to be a risk factor for coronary reconstruction (**Supplementary Table S2**, **Figures 3E, F**). We performed a subgroup analysis based on gender and age (age>60 or <=60) to further explore whether there were significant differences between the different subgroups. The results showed that semiquantitative scores remained risk factors for coronary reconstruction in all subgroups, and grade scores were risk factors in the subgroups of male, female and age > 60 years (**Supplementary Table S3**). However, we further tested interactions between age and grade scores in the regression model. The interaction terms were not statistically significant (*p*=0.198), indicating that the effect of grade scores on revascularization does not significantly vary by age.

**Table 6:** Multivariable logistic regression analysis for predictors of coronary reconstruction.

Variables	OR (95% CI)	*p*
HDL, mmol/L	0.31 (0.16-0.59)	<0.001
Semiquantitative scores	1.98 (1.65-2.47)	<0.001
Grade scores	1.95 (1.39-2.73)	<0.001

HDL, high-density lipoprotein.

## Discussion

Coronary atherosclerosis and peripheral atherosclerosis share similar pathophysiological mechanisms, involving systemic inflammation, endothelial dysfunction, and lipid accumulation (19). Thus, peripheral atherosclerosis is correlated with cardiovascular diseases. Atherosclerosis in different vascular regions can present distinct characteristics that reflect both local and systemic factors. Carotid and coronary arteries atherosclerosis often exhibit features of high-risk plaques, such as thin fibrous caps, large lipid cores, and inflammatory cell infiltration, which have been associated with embolic events leading to ischemic complications, including myocardial infarction (20). In contrast, lower limb atherosclerosis, typically seen in PAD, is frequently characterized by calcified and fibrotic plaques (21). This difference in plaque composition is thought to be influenced by variations in hemodynamic forces (such as shear stress) and regional differences in endothelial cell function (such as inflammatory and immune factors) (22). Carotid and lower limb atherosclerosis occur earlier than coronary artery atherosclerosis (23–25). Therefore, it is of great clinical significance to determine the severity of coronary artery lesions by evaluating carotid and lower limb atherosclerosis using a noninvasive ultrasound method. In this study, we applied two indices of carotid artery atherosclerosis and one composite index of lower limb atherosclerosis to determine coronary artery status and to quantify the necessity for coronary revascularization.

Our results demonstrated that the semiquantitative score and grade score from carotid artery ultrasound were positively correlated with the Gensini score of coronary artery lesions. Carotid plaque thickness and IMT are reliable indices for assessing the extent of coronary atherosclerosis. These results support previous studies which showed that the thickness of the largest plaque is an independent indicator of coronary events (26) and that IMT, the thickness of the largest plaque, and total carotid plaques increase with the severity of CHD (27). With the development of carotid plaques, the incidence of serious cardiovascular events such as CHD and myocardial infarction increases. The more severe the carotid plaque lesion, the greater the probability of plaque rupture, and the risk for plaque obstruction of the cardio-cerebrovascular arteries (28). Therefore, assessing the severity of carotid plaque lesions is helpful for preventing the incidence of cardiovascular events.

The group with higher vascular ultrasound scores in the lower limb had higher Gensini scores, indicating that the severity of lower limb atherosclerosis is related to the presence of coronary artery disease. A study in Spanish workers showed that the correlation between femoral atherosclerosis and coronary calcification (AUC of 0.719) was stronger than that between carotid artery atherosclerosis and coronary calcification (AUC of 0.589) (19). There is a significant correlation between carotid artery stenosis and the prevalence of lower limb artery disease (29). A 10-year follow-up study concluded that the risk of future cardiovascular events is significantly increased in patients with both carotid and lower limb atherosclerosis (30). Therefore, evaluating the blood vessels of the neck and lower limbs is a better indicator for CHD.

We quantified the necessity of coronary revascularization according to three indices of carotid artery and lower limb atherosclerosis. Based on the ROC curves derived from the integration method, the AUC of the grade score (0.82) was the highest, followed by the AUCs of the semiquantitative score (0.78) and lower limb score (0.72). A grade score of 2 distinguishes patients with lower plaque burden from those with higher plaque burden. When the grade score is 2, the specificity reaches 72.13%, indicating that this criterion is highly reliable in excluding low-risk patients. It helps identify patients who do not require revascularization, thereby reducing unnecessary invasive interventions and optimizing the allocation of medical resources. A lower limb score of 3 indicates that the patient’s lower limb arterial blood flow may already be compromised to some extent. This helps identify patients who require interventional therapy, reducing missed diagnoses. It is of great significance for the early detection of high-risk patients and timely intervention. Therefore, the combination of carotid artery grade score 2 and lower limb score 3 was the threshold for the prediction of coronary revascularization, with the highest sensitivity and specificity.

Additionally, due to similar pathophysiological mechanisms, such as hemodynamics, inflammatory and immune responses, as well as arterial diameter and wall thickness, patients with coexisting atherosclerosis in the carotid and lower limb arteries are at a higher risk of developing coronary artery disease. The presence of peripheral vascular-related complications, such as stroke, intermittent claudication, or amputation, further indicates the necessity for coronary revascularization. Ultrasound scoring systems for carotid and lower limb blood vessels can be particularly valuable for individuals at an intermediate to high risk of CHD, such as patients with risk factors including hypertension, diabetes, dyslipidemia, or a family history of cardiovascular disease. This data can serve as additional indicators for the risk of CHD, aiding clinicians in deciding on earlier preventive interventions. Higher carotid and lower limb scores derived from ultrasound imaging may indicate a greater burden of atherosclerosis and thus a higher likelihood of CHD. In clinical practice, patients with moderate to high scores could be prioritized for further testing (such as coronary angiography) and may benefit from more intensive lipid-lowering therapies, antihypertensive medications, or lifestyle interventions.

In recent years, clinical guidelines have suggested that atherosclerosis in lower limb and neck vessels reflects atherosclerosis in coronary arteries to a certain extent, but the methods for detection are not suitable for the primary screening for CHD (31–33). Computed tomography angiography (CTA) or magnetic resonance angiography (MRA) can clearly and accurately evaluate peripheral vascular disease, but it is expensive. On the other hand, ankle brachial index (ABI) is one of the most commonly used and simplest methods to assess peripheral blood vessels, but it lacks accuracy, and therefore is not reliable for diagnosis. In this study, we used relatively inexpensive and accurate vascular ultrasound to evaluate the degree of peripheral vascular stenosis and visualize the possible presence of atherosclerotic plaques. Peripheral vascular atherosclerosis was quantified by scoring to explore its predictive value for CHD, which is conducive to screening high-risk patients for coronary heart disease and evaluate their risk for myocardial infarction. This study sought to close the research gap in the relationship between peripheral blood vessels and CHD, and to develop a new, more accurate technique for the screening of CHD.

## Limitations

The study has several limitations. First, it was a single-center study with a limited sample size, which could reduce the statistical power. Future research should involve larger sample sizes and employ a more rigorous multicenter design to further validate our conclusions. Second, this study is retrospective, which relies on pre-existing data and may introduce an element of selection bias. Third, the lack of blinding in the ultrasound measurements performed by different sonographers may introduce inter-observer and intra-observer variability. Consequently, further prospective, randomized controlled trials are needed to confirm the quantitative correlation between carotid or lower limb atherosclerosis and CHD.

## Conclusions

In conclusion, we confirmed the positive correlation of carotid and lower limb atherosclerosis with the occurrence and severity of CHD. The carotid artery grade score and lower limb score from noninvasive ultrasound are promising indices for the early diagnosis of CHD and the prognosis following interventional therapy. To further explore the implications of our study, future research should consider a multicenter study to validate the correlation between carotid and lower limb atherosclerosis with CHD across diverse populations and healthcare settings. Expanding to a multicenter design would enhance the generalizability of these findings, address potential biases from single-center studies and provide a more comprehensive understanding of atherosclerosis patterns in CHD. Additionally, future studies should incorporate a broader range of biomarkers, including inflammatory markers and genetic predispositions, to assess their predictive value for CHD progression. Combining noninvasive imaging indices, such as carotid artery and lower limb scores, with molecular biomarkers could improve the accuracy of early diagnosis and offer more personalized assessments for CHD prognosis. This approach would provide a more robust, predictive model for guiding intervention strategies and monitoring disease progression in clinical practice.

## Data Availability

The original contributions presented in the study are included in the article/**Supplementary Material**. Further inquiries can be directed to the corresponding author/s.
