# Supplementary material for: Quantitative correlation between carotid or lower limb atherosclerosis and coronary heart disease: a retrospective observational study
Source: Front Endocrinol (Lausanne). 2025 Mar 24;16:1570942. doi: 10.3389/fendo.2025.1570942 (PMC11973080; doi:10.3389/fendo.2025.1570942)
Supplement: Supplementary file 1 [file DataSheet1.docx]

**Supporting material**

**Quantitative correlation between carotid or lower limb atherosclerosis and coronary heart disease: a retrospective observational study**

Zeyu Jiang^1†^, Shimiao Ruan^2†^, Kun Zhao^3†^, Shuhan Pan^2*^, Wenzhong Zhang^1*^

^1^ Department of Cardiology, The Affiliated Hospital of Qingdao University, Qingdao 266021, China

^2^ Department of Emergency Medicine, The Affiliated Hospital of Qingdao University, Qingdao 266021, China

^3^ Department of Cardiology Medicine, Qingdao Central Hospital, Qingdao 266021, China

*Correspondence: xxmczwz@163.com (Wenzhong Zhang), panshe88@126.com (Shuhan Pan)

^†^These authors contributed equally.

**Table S1.** Semiquantitative scores, grade scores and lower limb scores

| **Semiquantitative scores** [12] | | | |
| --- | --- | --- | --- |
| **Scores** | **Standard** | | |
| 0 | normal, no intimal thickening, IMT < 1.0 mm | | |
| 1 | the intimal is thickened locally, IMT < 1.2 mm | | |
| 2 | arteriosclerotic plaque formed, but no significant stenosis | | |
| 3 | stenosis rate above 25% | | |
| **Grade scores** [13] | | | |
| **Scores** | **Standard** | | |
| 0 | no plaque; | | |
| 1 | 1 plaque with thickness ≤ 2.0 mm | | |
| 2 | 2 plaques with thickness < 2.0 mm or 1 plaque with thickness > 2.0 mm | | |
| 3 | 2 plaques with thickness > 2.0 mm | | |
| 4 | more than 2 plaques with thickness > 2.0 mm | | |
| **Lower limb scores** [14]: based on the pooled scores of the following three factors | | | |
| **Scores** | **Standard** | | |
|  | (1) Intimal thickness | (2) Plaque | (3) Stenosis |
| 0 | < 1.0 mm | without plaque | no stenosis |
| 1 | 1.0-1.2 mm | single occurrence | 30-50% stenosis ratio |
| 2 | > 1.2 mm | multiple occurrence | 50-75% stenosis ratio |

**Table S2.** Univariate logistic regression analysis for predictors of coronary reconstruction

| Variables | OR (95% CI) | *p*-value |
| --- | --- | --- |
| Female | 1.29 (0.81-2.09) | 0.285 |
| Diabetes | 1.31 (1.18-1.51) | 0.201 |
| Hypertension | 1.03 (0.64-1.64) | 0.911 |
| Smoking | 0.85 (0.52-1.38) | 0.524 |
| Drinking | 1.27 (0.93-1.54) | 0.126 |
| Age, years | 1.02 (0.99-1.05) | 0.089 |
| BMI, kg/m2 | 1.04 (0.96-1.14) | 0.323 |
| TC, mmol/L | 0.82 (0.66-1.03) | 0.092 |
| TG, mmol/L | 1.11 (0.83-1.48) | 0.486 |
| HDL, mmol/L | 0.28 (0.16-0.49) | <0.001 |
| LDL, mmol/L | 1.09 (0.81-1.47) | 0.563 |
| Semiquantitative scores | 1.79 (1.45-2.23) | <0.001 |
| Grade scores | 1.71 (1.46-2.02) | <0.001 |
| Lower limb scores | 1.29 (1.12-1.79) | 0.037 |

**Table S3.** Subgroup analysis by age and gender

| Variables | OR (95% CI) | p-value |
| --- | --- | --- |
| Age > 60 |  |  |
| HDL, mmol/L | 0.34 (0.16-0.71) | 0.004 |
| Semiquantitative scores | 1.56 (1.58-2.43) | 0.035 |
| Grade scores | 1.91 (1.32-2.78) | 0.001 |
| Age ≤ 60 |  |  |
| HDL, mmol/L | 0.19 (0.04-0.85) | 0.03 |
| Semiquantitative scores | 2.11 (1.38-3.21) | 0.039 |
| Grade scores | 2.26 (0.98-5.25) | 0.056 |
| Male |  |  |
| HDL, mmol/L | 0.16 (0.04-0.61) | 0.007 |
| Semiquantitative scores | 1.17 (1.09-2.4) | 0.017 |
| Grade scores | 1.78 (1.01-3.1) | 0.004 |
| Female |  |  |
| HDL, mmol/L | 0.38 (0.17-0.92) | 0.014 |
| Semiquantitative scores | 1.89 (1.52-2.54) | 0.032 |
| Grade scores | 2.01 (1.27-3.19) | 0.003 |


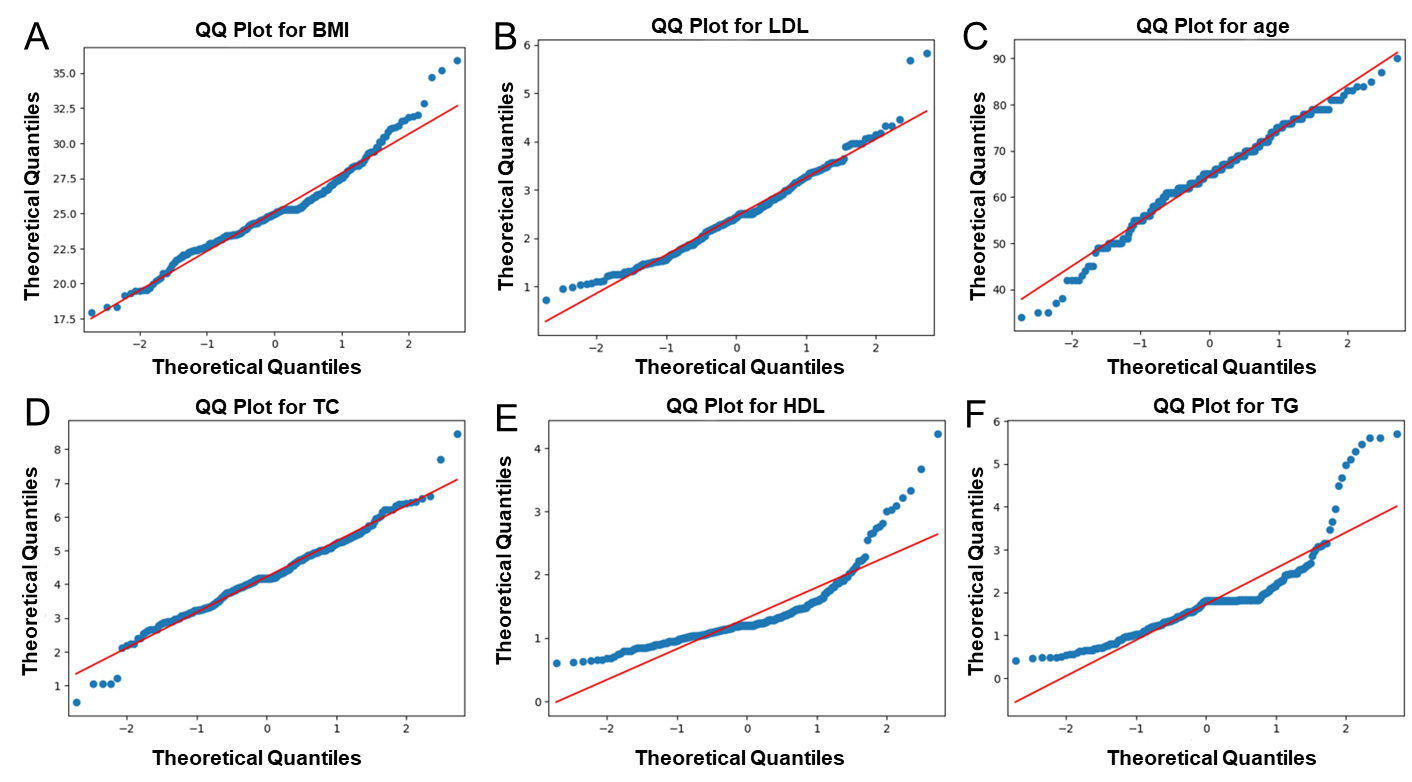


**Figure S1** QQ plot for test the normal distribution of continuous variables. (A) BMI, (B) LDL, (C) age, (D) TC, (E) HDL and (F) TG.


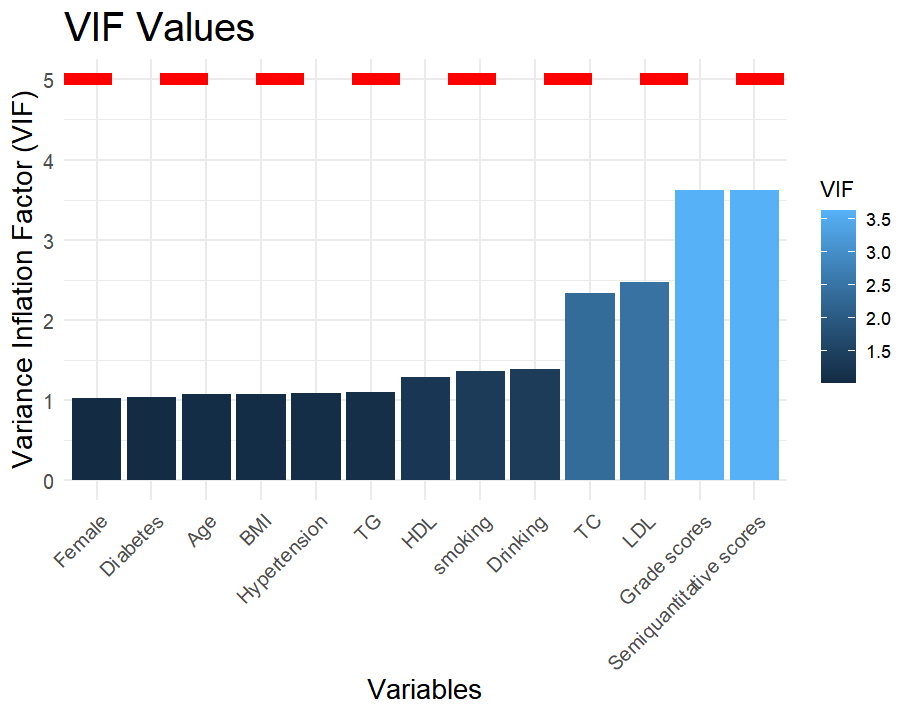


**Figure S2** Bar Plot of VIF values for predictor variables.


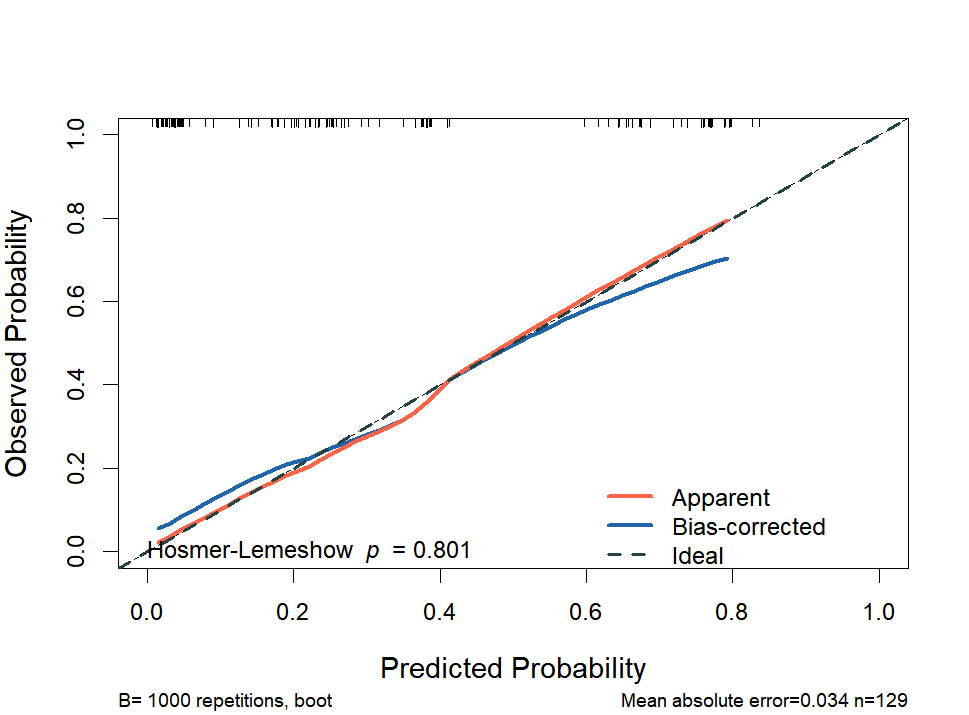


**Figure S3** Calibration plot of multivariable logistic regression model.
